# Boldness suppresses hoarding behavior in food hoarding season and reduces over‐wintering survival in a social rodent

**DOI:** 10.1002/ece3.11252

**Published:** 2024-04-09

**Authors:** Lin Gan, Shuang‐Jie Tian, De‐Hua Wang, Wei Liu

**Affiliations:** ^1^ State Key Laboratory of Integrated Management of Pest Insects and Rodents, Institute of Zoology Chinese Academy of Sciences Beijing China; ^2^ Jiangsu Key Laboratory for Biodiversity and Biotechnology, College of Life Sciences Nanjing Normal University Nanjing China; ^3^ University of Chinese Academy of Sciences Beijing China; ^4^ CAS Center for Excellence in Biotic Interactions University of Chinese Academy of Sciences Beijing China; ^5^ School of Life Sciences Shandong University Qingdao China

**Keywords:** animal personality, food hoarding, *Meriones unguiculatus*, over‐wintering adaptation, pace‐of‐life syndrome

## Abstract

The “pace‐of‐life” syndrome (POLS) framework can encompass multiple personality axes that drive important functional behaviors (e.g., foraging behavior) and that co‐vary with multiple life history traits. Food hoarding is an adaptive behavior important for an animal's ability to adapt to seasonal fluctuations in food availability. However, the empirical evidence for the relationships between animal personality and hoarding behavior remains unclear, including its fitness consequences in the POLS framework. In this study, the Mongolian gerbil (*Meriones unguiculatus*), a social rodent, was used as a model system to investigate how boldness or shyness is associated with food hoarding strategies during the food hoarding season and their impact on over‐winter survival and reproduction at both individual and group levels. The results of this study showed that, compared with shy gerbils, bold gerbils had a lower effort foraging strategy during the food hoarding season and exhibited lower over‐winter survival rates. However, bold–shy personality differences had no effect on over‐winter reproduction. These findings suggest that the personality is a crucial factor influencing the foraging strategy during the food hoarding season in Mongolian gerbils. Personality may be related to energy states or the reaction to environmental changes (e.g., predation risk and food availability) in bold or shy social animals. These results reflect animal life history trade‐offs between current versus future reproduction and reproduction versus self‐maintenance, thereby helping Mongolian gerbils in adapting to seasonal fluctuations in their habitat.

## Graphical abstract

Our research illustrates that boldness could suppress food hoarding behavior and over‐wintering survival in Mongolian gerbils (*Meriones unguiculatus*) at both individual and group levels, and suggests that personality was a key factor that affected the foraging strategy during the food hoarding season, and may be affected by their life history trade‐offs.

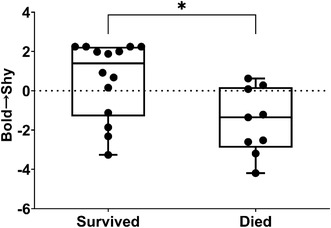

## 1 INTRODUCTION

Foraging, including finding food, feeding, and storage, is a fundamental behavior for that ensures the survival and reproduction of animals (Stephens & Krebs, 1986). Individuals vary in terms of how they acquire food, and foraging decisions could be state‐dependent on an animal's current physiological state (e.g., hunger), as well as the current environmental state (e.g., actual risk of predation and food availability). Moreover, these decisions may also be based on past and expected future states and risks, thereby facilitating the optimal trade‐off between energetic gain from foraging and cost due to risks (Luttbeg & Sih, 2010). Hoarding behavior, which differs from consummatory behavior (e.g., feeding), is an appetitive behavior for animals living in an environment with unpredictable food availability (Bronson, 1989; Pravosudov & Clayton, 2002). Some animals have evolved to utilize food hoarding behavior to control the availability of food in space and time to improve their chances of survival (Vander Wall, 1990). Hoarding behavior variations may be explained by frequency‐dependent selection (Roff, 1998), but they may also arise from variations in personality, which may reflect alternative strategies with environmentally dependent adaptive value (Dall et al., 2004; Sih et al., 2004).

Recently, considerable progress has been made in understanding the ecological and evolutionary basis for variations in animal personality (Planas‐Sitjà et al., 2015; Quinn, 2015; Vanden Broecke et al., 2021). Personality has been predicted to drive important functional behaviors that can influence life history strategies (Bolnick et al., 2003; Dall et al., 2004; Réale et al., 2010; Sih et al., 2004; Wolf & Weissing, 2010). The “pace‐of‐life” syndrome (POLS) theory predicts that variations in behavior may be maintained because suites of phenotypic characteristics (e.g., morphological, physiological, and life history) covary to balance trade‐offs between current and future reproduction, allowing individuals with different life history strategies to achieve similar fitness in the same time and place (Réale et al., 2010; Stamps, 2007; Wolf et al., 2007). Thus, variations in animal personality and related behavioral differences can be explained by trade‐offs between current and future reproduction/survival. Reproduction necessitates a substantial expenditure of energy; however, individuals typically face limitations in their energy acquisition. Consequently, individuals must make trade‐offs in their energy allocation. Some individuals prioritizing current reproduction over survival, while others invest more in survival than reproduction. This individual variation may generate differences in the life history strategies of individuals (Réale et al., 2010; Wolf & Weissing, 2010), which are mediated by effects of ecological variables on adaptive state‐behavior feedbacks (Luttbeg & Sih, 2010; Sih et al., 2015).

Indeed, several studies have shown that personality can predict an individual's foraging behavior when faced with the risk of predation (Arnold et al., 2007; Quinn et al., 2012; van Oers et al., 2004), as well as competitive foraging behavior (Cole & Quinn, 2012; David et al., 2011; Riebli et al., 2011) and foraging flexibility (Verbeek et al., 1994). For example, highly aggressive convict cichlids (*Amatitlania nigrofasciata*) prefer complex habitats and tend to forage more frequently in open habitats, even in high‐predator contexts (Church & Grant, 2019). Unlike foraging behavior, food hoarding behavior is more crucial for future adaptation in some animals. Although some studies discuss the correlation between personality and food hoarding behavior in mammals. Most research focuses on scatter‐hoarding species and their ecological functions (Brehm & Mortelliti, 2022; Zwolak, 2018). The relationship between personality and hoarding behavior in larder‐hoarding species, as well as its fitness consequences, are still rarely discussed. Furthermore, some research has shown that individual fitness is related to their personalities, as demonstrated in studies of black‐browed albatross (*Thalassarche melanophrys*), where bold individuals exhibit a higher reproductive rate (Patrick & Weimerskirch, 2014). However, a meta‐analysis revealed that the relationships between personality, behaviors, and life histories were not always consistent (Moiron et al., 2020; Royauté et al., 2018). This inconsistency may be reflected in the environment; the fluctuation of the environment may play a critical role in these results. Indeed, a recent study showed that in a safety context, more exploratory lemon sharks (*Negaprion brevirostris*) in captivity were also more willing to take risks in the wild and grew faster with lower apparent survival, but in a predator‐rich context, the link between exploratory personality and the growth‐mortality trade‐off disappeared. Another study on the great tit (*Parus major*) also demonstrated that the correlation between personality and adaptation is opposite between years with high winter food abundance and years with winter food shortages (Dingemanse et al., 2004). Together, these results show that the association between personality and life history is favored in some ecological contexts but not in others (Dhellemmes et al., 2021). Thus, the local ecological context could play an important role in shaping and maintaining trait correlations. In particular, non‐hibernating animals may face food shortages and increased energy requirements during the winter (Jackson et al., 2001; Merritt, 1995; Wang & Wang, 1996). This potential imbalance in food supply and requirements could have led to the evolution of features for coping with winter conditions, such as hoarding food (Nyby & Thiessen, 1980). However, the correlation between personality‐driven food hoarding behavior and its fitness consequences (e.g., life history trade‐offs) has rarely been investigated in non‐hibernating mammals.

Mongolian gerbils (*Meriones unguiculatus*) are geographically widespread in steppe, semi‐desert, and desert habitats in northern China, southeast Mongolia, and the southern TransBaikal and south of the Tuva region in Russia (Batsaikhan & Tsytsulina, 2016; Wilson & Reeder, 2005). Mongolian gerbils live in family groups of 2–18 individuals (Agren et al., 1989a, 1989b; Liu, Wang, Wan, & Zhong, 2009; Liu, Wang, Wang, et al., 2009; Wang et al., 2011). In the field, each social group excavates and occupies a complex burrow system. This system comprises underground feeding chambers, nest chambers, tunnels, and aboveground entrances and trails for reproduction, storing food, and escaping from both predators and environmental stress (Scheibler et al., 2006; Wang et al., 2011). Burrow colonies, or cave groups, encompass all entrances and trails of the same burrow system, and the home range of a burrow colony is approximately 309.10 m^2^ (Wang et al., 2011; Wang & Zhong, 1998). Reproduction by Mongolian gerbils mainly occurs from March to August, with a breeding lull in the autumn and winter (only 10% of gerbils are reproductively active) (Liu et al., 2007; Liu, Wang, Wan, & Zhong, 2009; Liu, Wang, Wang, et al., 2009). Mongolian gerbils are non‐hibernating rodents, and thus, they are typically influenced by seasonal fluctuations in food availability (Agren et al., 1989a, 1989b). Gerbils maximize their chances of surviving the winter by storing relatively large amounts of food (Hsia & Wang, 1956; Nyby & Thiessen, 1980). In the wild, food hoarding is a cooperative behavior in Mongolian gerbils. All group members participate in food hoarding, with some individuals primarily responsible for locating food, chasing strangers, or defending their resources, while others dedicate their time to transferring the hoarded food to food patches within the burrow system (Agren et al., 1989b; Liu et al., 2005). Previous studies have shown that gerbils exhibit consistent individual differences in their food hoarding behavior; some individuals exhibit a higher hoarding tendency while others show lower or no hoarding behavior, but no sex differences (Nyby et al., 1973; Nyby & Thiessen, 1980). However, the reasons for these individual variations have not yet been fully investigated.

In this study, we investigated the interplay between boldness, hoarding behavior, and over‐wintering reproduction and survival in semi‐natural populations of Mongolian gerbils as an empirical test of the POLS hypothesis. We tested how hoarding behaviors are related to personality at the individual and group levels, as well as how personality is associated with over‐wintering survival and reproduction at the group level. Hoarding behavior is distinct from feeding behavior and is acknowledged as an investment in future fitness. According to the POLS hypothesis, bold individuals are expected to have higher reproduction than shy ones, but shy individuals are expected to have a longer lifespan than bold ones. Hence, we predicted that shyer gerbils, with a longer expected lifespan, would invest more in foraging and hoarding, and have a higher over‐winerting survival rate.

## 2 MATERIALS AND METHODS

### 2.1 Animal collection and maintenance

Gerbils were collected along roads in Suniteyouqi County (41°55′ N, 111°08′ E) in Inner Mongolia during May 2019. We placed one or two wire‐mesh live traps (28 × 13 × 10 cm) with a peanut at each trap station, with the trap door open and facing an active gerbil entrance or runway to maximize the probability of capture (Liu et al., 2007). Trapping session were start at 5:00 a.m. to 10:00 a.m. and 15:00 p.m. to 20:00 p.m. each day. We checked traps every hour and moved captured gerbils into a cage (46 × 30 × 18 cm) for avian gerbils dying from overexposure to sunlight. Then, 192 sub‐adult or adult (96 males and 96 non‐pregnant females) gerbils were transported back to Taipusi Qi Field Research Station in Inner Mongolia, China (41°58′ N, 115°17′ E; 1500 m elevation) where the study was conducted. The area of the study station was typical steppe intermixed with cropland. The climate of the study area was semiarid and continental, with a relatively hot summer and a cold, dry winter, that is, with an average monthly temperature range of −19.1°C to 21°C and an annual total precipitation of approximately 350 mm, where snow occurred from mid‐ to late‐October until early April (Liu, Wang, Wan, & Zhong, 2009; Liu, Wang, Wang, et al., 2009).

After transport, all gerbils were housed in the field research station under laboratory conditions with natural light and room temperature. Gerbils had free access to sufficient water and food (commercial standard rat pellets, KeAo Bioscience Co., Beijing). All gerbils were tagged with an radio‐frequency identification (RFID, Raybaca IOT Technology, Beijing) for individual identity. Then, according to the average number of seven gerbils per burrow system in the field (Agren et al., 1989a, 1989b; Liu, Wang, Wan, & Zhong, 2009; Liu, Wang, Wang, et al., 2009), eight gerbils (four males and four females), most likely using the same wild gerbil group burrow systems or home ranges (i.e., wild original groups), were placed in an experimental social group and housed in a cage (46 × 30 × 18 cm) by which it is likelihood to increase males and females forming a social group. After 4 weeks of group acclimation, we investigated the boldness of individuals and then randomly chose eight social groups where the gerbils had similar body size for our semi‐natural foraging behavior study. The rest of the experimental groups (16 groups) were transferred to laboratory conditions for other studies.

### 2.2 Boldness measurement

The boldness of each gerbil was measured in an elevated plus maze (EPM) according to the procedures used for other rodents during the light period (Carobrez & Bertoglio, 2005; Vobrubová et al., 2017) when they were housed under laboratory conditions in July 2019. Previous studies showed that the number of entries, moving distance, and time spent in the open arms can be used to assess the boldness of individuals (Améndola et al., 2022; Rudolfová et al., 2022), and other studies have suggested that the percentage of entries, percentage of moving distance, and percentage of time spent in the open arms also reflect the boldness of individuals (Lister, 1987; Nieminen et al., 1992; Pellow et al., 1985; Reichard et al., 2019). Thus, we referenced the previous studies and recorded six behavioral parameters: entries in the open arms; time spent in the open arms (s); distance moved in the open arms (cm); a ratio of open arm entries to the closed arm entries (ROE); a ratio of time spent in open arms relative to time spent in the closed arms (ROT); and ratio of distance moved to open arms relative to distance moved to the closed arms (ROM) to assessed gerbils' boldness behavior. All individuals completed the EPM test twice to assess the repeatability of the bold‐shy personality results, and the tests were separated by 1 week. The EPM apparatus comprised two closed arms with dimensions of 42.5 × 10 × 30 cm and two open arms with dimensions of 46.5 × 10 cm, and it was positioned 70 cm above the floor (Zhenghua Biology, Anhui). First, we placed individual gerbils at the hub where the open and closed arms crossed and let the gerbil face a closed arm before video recording for 5 min. After each session, any feces were removed from the EPM and the floor of the maze was wiped with 75% alcohol to remove any urine or scent cues. Video analyses were performed using EthoVision XT (Noldus, Netherlands).

### 2.3 Field foraging behavior

**FIGURE 1:**
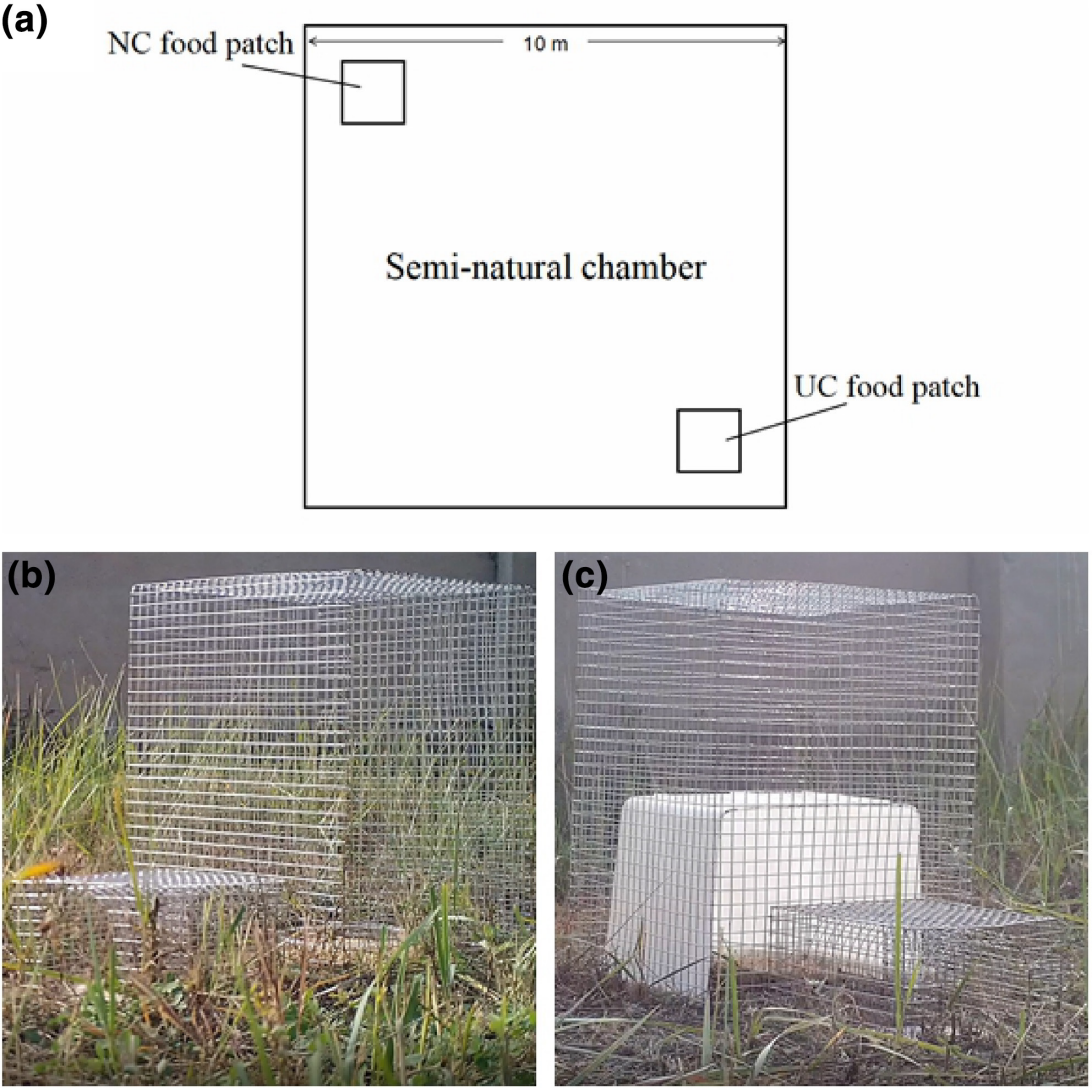
(a) Schematic illustration of the semi‐natural sub‐chambers. (b) No cover (NC) food patch. (c) Under cover (UC) food patch.

We released the eight selected groups, which had acclimated for 4 weeks in the laboratory before the field foraging behavior experiments, into our semi‐natural enclosures. The enclosure consisted of eight sub‐chambers (each measuring 10 × 10 m), respectively. The outer enclosure and sub‐chambers were constructed of cement walls situated approximately 100 cm above the ground and 100 cm below the ground and no connections between neighboring sub‐chambers, to prevent the escape of the gerbils or the entry of other sub‐chamber burrowing rodents. The sides and top of the enclosure was covered with nylon netting to prevent terrestrial and avian predation (e.g., *Bubo bubo*) (Liu et al., 2011; Liu, Wang, Wan, & Zhong, 2009; Liu, Wang, Wang, et al., 2009). Each of the eight groups was acclimated in a separate sub‐chamber for 5 weeks and fed on natural grasses and herbs (e.g., *Potentilla* spp., *Heteropappus altaicus*, *Serratula centauroides*, and *Leymus chinensis*), and we randomly placed three quadrats measuring 1 × 1 m in each sub‐chamber and cut all plants at the ground level within a sampling quadrat, before weighing the fresh biomass (grams per square meter) in these enclosures. The average standing fresh plant biomass was about 457.3 g/m^2^ (SE = 22.3) at the end of August. According to a previous study, the fresh plant biomass was 6.2–139.5 g/m^2^ in a wild gerbil habitat (Agren et al., 1989a, 1989b). Mongolian gerbils consume approximately 5.5 g of wheat per adult per day (Hsia & Wang, 1956). Thus, all of these plant resources in each sub‐chamber were similar and sufficient for group gerbils over a 5‐month period, except the difference wheat seeds storage in each group during the hoarding behavior experiments. The experimental sub‐chamber (100 m^2^) was about 20 times larger than the core area (4.1 m^2^) of a gerbil burrow system (Hsia & Wang, 1956). In mid‐September 2019 (i.e., 5 weeks after release), we detected a total of 24 gerbils in eight groups with 2–6 individuals in each group according to RFID record data and recaptured data after hoarding behavior experiment. Then, we started the field foraging behavior experiments. Gerbils in each social group were observed in their sub‐chambers under different food patch treatments (Figure 1a): “no cover” (NC) patch (in an open field without a cover; Figure 1b) and “under cover” (UC) patch (in an open field with an artificial cover measuring 25 × 25 × 18 cm; Figure 1c).

We placed a foraging tray in each patch, which comprised a glass tray (18 cm in diameter) buried in the dirt and 150 g of wheat grains were placed in the tray. The UC food patches were protected from natural foraging birds (e.g., *Passer montanus*) by heavy wire frames and fine filament fish netting with a passageway measuring 10 × 10 × 20 cm. Gerbils readily reached the trays by passing through the passageway, and individual foraging activity was recorded by an RFID tag reader (RBC‐S03; Raybaca IOT Technology, Beijing), which was buried in the soil under each food patch passageway. When a marked gerbil entered or exited a patch, its RFID tag identification code was logged with the exact time of entry or exit. Food patches were replenished at dawn and dusk. We retrieved all of the remaining wheat grains and weighed them 2 h after they were replenished. We conducted six replicate of experiment for each group (two times per day for three consecutive days) from 6:00 to 8:00 and 16:00 to 18:00, and changed the position of the NC food patch and UC food patch every day to exclude the effects of positional factors on the experiment. We recorded the time to first foraging (foraging latency), number of foraging bouts (foraging frequency), and duration of each foraging bout performed by each gerbil. We also calculated the group foraging efficiency (the average amount of hoarded wheat (in g) by all individuals within a group) and used the average foraging frequency of all individuals within a group to calculate the group foraging frequency.

### 2.4 Over‐wintering reproduction and survival

In late April 2020, we recaptured all of the gerbils in the enclosures by placing wire‐mesh live traps at gerbil burrow endted for 3–5 consecutive days (Liu et al., 2007). We scanned the RFID tags to identify the surviving individuals and used the ratio of the number recaptured relative to the number recorded in each group in mid‐September 2019 to assess the over‐wintering survival by groups. In addition, we counted the number of gerbils without RFID tags and then calculated the over‐wintering reproduction rate; that is, the ratio of the number of new juvenile or sub‐adult gerbils relative to the number of adult females (recaptured in late April 2020) in each group.

### 2.5 Statistical analysis

#### 2.5.1 Boldness

We used the methods described by Rupia et al. (2016) to analyze individual boldness. Instead of exploring the separate correlations among scores for each behavioral parameter, we conducted a principal component analysis (PCA; using the “ade4” package in R) to obtain composite personality scores for each individual based on six performance parameters collected during the first boldness assay (Wauters et al., 2021). We then imported the second boldness assay data into the PCA model to obtain the second boldness score and used a Markov chain Monte Carlo (MCMC) generalized linear mixed model approach (“MCMCglmm” package in R) to assess the repeatability (Hadfield, 2010). We calculated the repeatability of the boldness scores using linear mixed models with experiment number as a fixed effect, gerbil ID and groupID as a random effect. We calculated 95% credible intervals (95% CIs) to assess the repeatability by running 1000 permutations of each test and using the posterior distributions to calculate the repeatability as: *R* = *V*_ind_/(*V*_ind_ + *V*_e_). We used the first PCA result as a personality score and used an independent *t*‐test to compare the boldness scores of male and female individuals. In order to better assess the group personality, especially group composition, effects on gerbils' behavior and over‐wintering adaptation. We used a dissimilarity index (“vegan” package in R) and Ward's hierarchical clustering method (“stats” package in R) to categorize gerbils into bold and shy behavioral types (Rupia et al., 2016). The average boldness score, derived from all individuals within a group, was considered the group personality score (Farine et al., 2015; Vágási et al., 2021), while the proportion of bold individuals in the group was used to determine the group composition.

#### 2.5.2 Foraging behavior

To analyze the factors that influenced foraging behavior at an individual level, we used a MCMC generalized linear mixed model (“MCMCglmm” package in R) to determine the effects of independent variables on individual hoarding behavior, and running a separate model for each hoarding behavior types, with boldness types, food patch type, and sex as fixed effects and gerbil ID, food patch locations, and experiment conduct times as random effects. Group foraging behavior was also analyzed in the MCMC generalized linear mixed model, and running a separate model for foraging frequency and food hoarding weight, with the boldness score, group composition (ratio of shy individuals and group members during hoarding experiment), and food patch type as fixed effects and sub‐chamber ID, group member numbers, food patch locations, and experiment conduct times as random effects. All the MCMC molds were ran for 13,000 interactions with 3000 burn‐in phase of iterations and a thinning interval of 10.

#### 2.5.3 Over‐wintering survival and reproduction

Survival is binomially distributed, so we used logistic regression to test the significance of individual survival between bold and shy individuals (Réale & Festa‐Bianchet, 2003). At the group level, the Spearman's correlation coefficients were calculated to analyze the associations between boldness score and over‐wintering survival, reproduction, survival of bold group members, and survival of shy group members, as well as the associations between group composition and over‐wintering survival, reproduction, survival of bold group members, and survival of shy group members. All the analyses were conduct by IBM SPSS Statistics 26.0, and all data were expressed as the mean ± standard error of the mean (SEM). *p* < .05 was considered to indicate a statistically significant difference.

### 2.6 Ethical note

All experiments complied with the ASAB/ABS Guidelines for the Use of Animals in Research and they were approved by the Institutional Animal Use and Care Committee of the Institute of Zoology, Chinese Academy of Sciences (Ethical Inspection License No: IOZ13047).

## 3 RESULTS

### 3.1 Boldness in Mongolian gerbils

PCA reduced the number of boldness variables to two components (Table 1), which together explained 92.72% of the total variance in boldness. The first component (PC1) explained 85.63% of the variance, and the second component (PC2) explained 7.09% of the total variance. PC1 was the only component with an eigenvalue >1, and thus the transformed data for PC1 were subsequently used as a proxy for the boldness scores. Gerbils were clustered into two distinct groups along the shy‐bold continuum based on their personality scores, which bold gerbils scores were −2.36 ± 1.84 and shy gerbils scores were 1.48 ± 0.77. And the independent *t*‐test did not find a significant difference in boldness score between male (0.16 ± 0.22) and female (−0.16 ± 0.24) gerbils (*t* = 0.958, *p* = .339). Repeatability analysis showed that the boldness scores obtained for Mongolian gerbils were highly repeatable [*r* = .714, 95% CI (0.629, 0.773)], thereby indicating consistent between‐individual differences in boldness over time.

**TABLE 1:** Eigenvalues and eigenvectors of the first two components (PC1 and PC2) representing the percentage explained variance.

Parameters	Component 1 (PC1)	Component 2 (PC2)
Entries in the open arms	−0.3875	−0.5832
Time spent in the open arms	−0.4247	0.1683
Moving distance in the open arms	−0.4214	0.1601
ROE	−0.3850	−0.5679
ROT	−0.4090	0.4406
ROM	−0.4200	0.2988
Eigenvalue	5.14	0.43
Total variance (%)	85.63	7.09

Abbreviations: ROE, ratio of open arm entries relative to closed arm entries; ROM, ratio of distance moved to open arms relative to distance moved to closed arms; ROT, ratio of time spent on open arms relative to time spent on closed arms.

### 3.2 Boldness and individual foraging behavior

**FIGURE 2:**
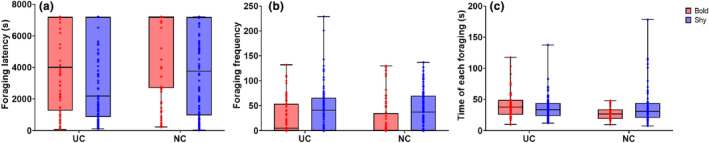
Foraging behavior of bold and shy gerbils in under cover (UC) and no cover (NC) food patch types. (a) Foraging latency. (b) Foraging frequency. (c) Duration of each foraging bout.

Individual foraging latency analysis showed that bold gerbils started foraging later than shy ones [*β* = −2461.0, 95% CI (−4351.9, −695.8), *p*MCMC = .008; Figure 2a, Table S1], and both bold and shy gerbils started foraging later in the NC food patch [*β* = −1634.9, 95% CI (−2794.1, −505.0), *p*MCMC = .014; Figure 2a, Table S1]. Foraging frequency analysis based on the MCMCglmm results showed that bold individuals foraged less frequently than shy individuals [*β* = 32.07, 95% CI (8.88, 56.18), *p*MCMC = .008; Figure 2b, Table S1]. Analysis of the duration of each foraging bout based on the MCMCglmm results suggested that bold gerbils had shorter foraging bouts than shy ones [*β* = 15.57, 95% CI (2.67, 31.56), *p*MCMC = .040; Figure 2c, Table S1], and gerbils spent less time in the NC food patch compared with the UC food patch [*β* = 15.47, 95% CI (0.71, 30.97), *p*MCMC = .038; Figure 2c, Table S1]. In addition, we found a significant interaction between boldness and food patch type with respect to the duration of each foraging bout [*β* = −23.27, 95% CI (−41.56, −5.18), *p*MCMC = .012; Figure 2c, Table S1]. The three foraging behavior measures did not differ significantly between males and females (Table S1).

### 3.3 Group boldness and foraging behavior

Analysis of the foraging frequency in the group level showed that bold groups foraged less frequently than shy groups [*β* = 14.16, 95% CI (8.12, 20.48), *p*MCMC < .001, Table 2]. Bold groups also hoarded lower food weights than shy groups [*β* = 10.28, 95% CI (7.10, 13.85), *p*MCMC < .001; Table 2]. However, the group foraging frequency and food hoard weight did not differ significantly between food patch types and group compositions (Table 2).

**TABLE 2:** Effects of group (*n* = 8) boldness (boldness score) and food patch type (UC and NC) on foraging behavior (frequency and food hoard weight) of Mongolian gerbils (*Meriones unguiculatus*) observed in field enclosures from mid‐September 2019 to mid‐October 2019 in Taibusi Qi, Inner Mongolia, China.

Behavior type	Posterior mean	Lower 95% CI	Upper 95% CI	*p*MCMC
Foraging frequency				
(Intercept)	**31.74**	**−0.52**	**53.95**	**.038**
Boldness score	**14.16**	**8.12**	**20.48**	**<.001**
Food patch_UC_	0.54	−23.10	24.72	.984
Group composition	15.99	−13.84	41.77	.232
Boldness score × Food patch_UC_	−1.46	−9.82	6.85	.726
Group composition × Food patch_UC_	5.79	−30.46	41.74	.752
Food hoarding weight				
(Intercept)	21.09	0.30	59.83	.058
Boldness score	**10.28**	**7.10**	**13.85**	**<.001**
Food patch_UC_	2.49	−9.97	15.61	.750
Group composition	8.10	−8.16	22.67	.292
Boldness score × Food patch_UC_	−2.10	−6.30	3.00	.354
Group composition × Food patch_UC_	0.90	−20.00	20.04	.922

*Note*: Group composition represents the proportion of bold gerbils in the group. Posterior means, 95% confidence intervals, and probability values (*p*MCMC) are presented. Model estimates are shown in bold when the confidence intervals do not overlap 0.

### 3.4 Over‐wintering survival and reproduction

**FIGURE 3:**
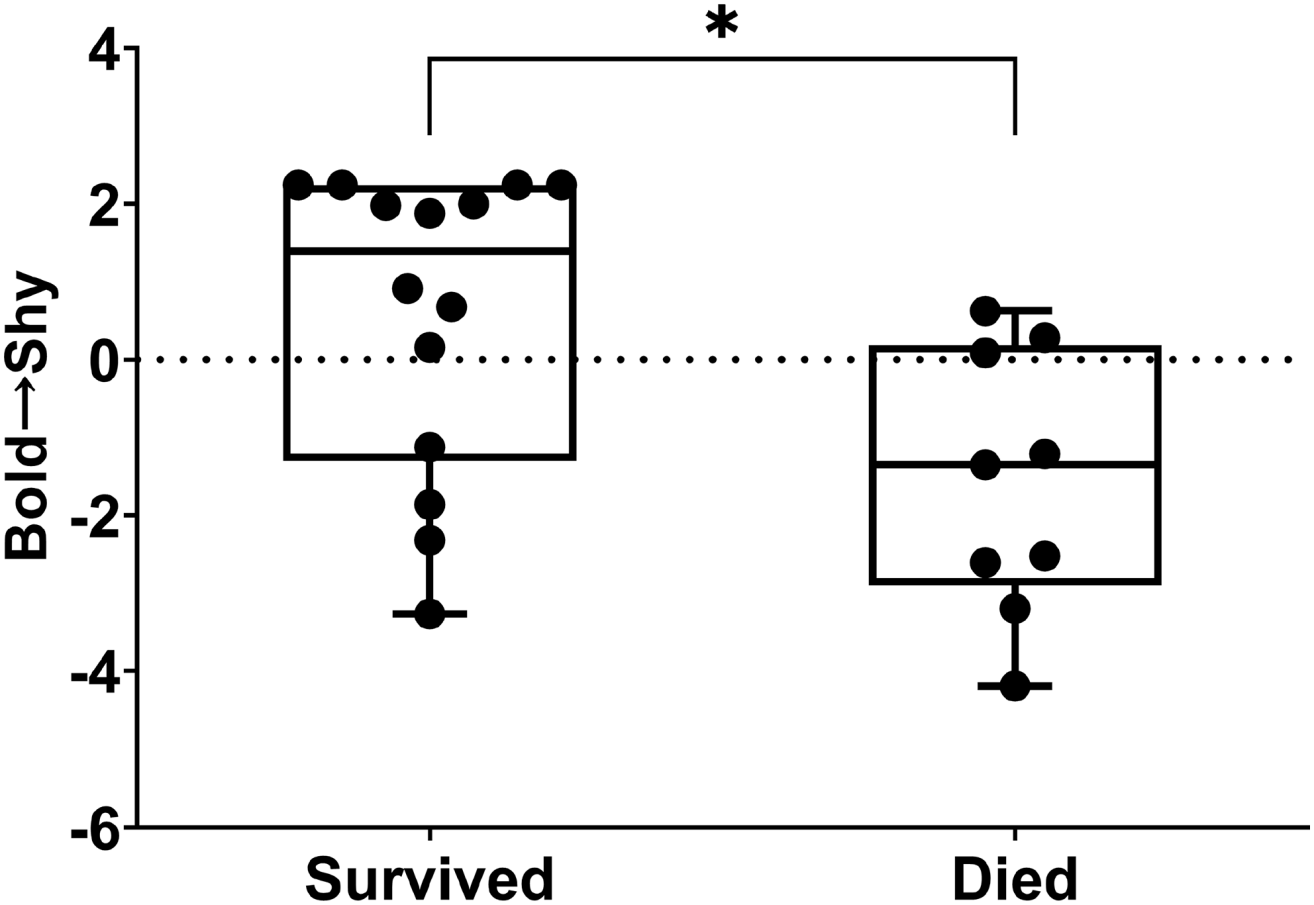
Boldness score as a function of apparent over‐wintering survival in Mongolian gerbils (*n* = 24). *p < .05.

**FIGURE 4:**
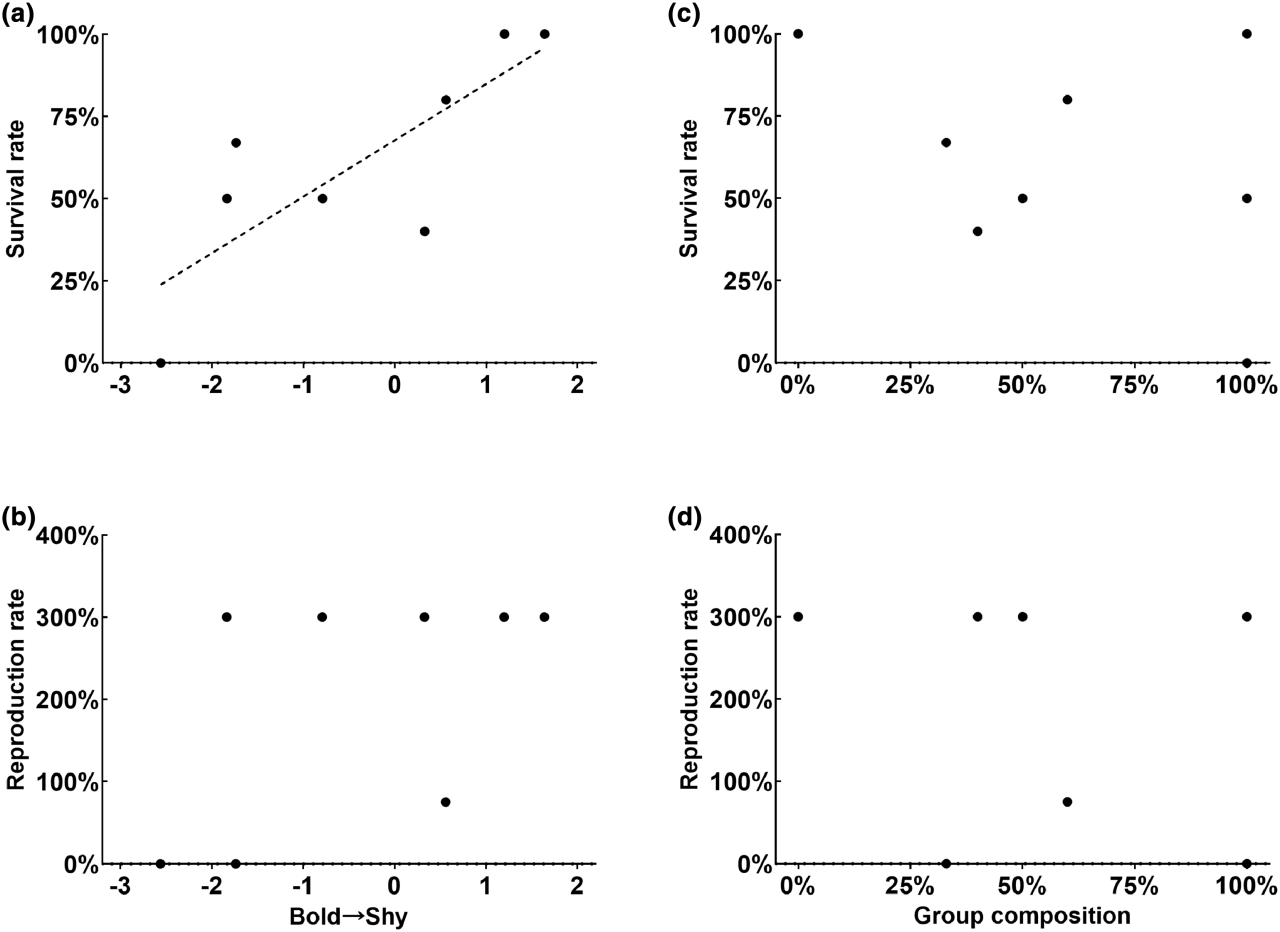
Analysis based on Spearman's correlation coefficients between: (a) group boldness score and over‐wintering survival; (b) group boldness score and over‐wintering reproduction; (c) group composition and over‐wintering survival; and (d) group composition and over‐wintering reproduction. “Group boldness score” represents the average boldness score of all group members. “Group composition” represents the proportion of bold gerbils in the group. The dashed lines represent the regression lines fitted to the data.

In April 2020, we recaptured all the gerbils of each groups to assess their over‐wintering survival and reproduction (Table S2). At the individual level, shy individuals had a greater probability of surviving over the winter (odds ratio [OR] = 1.81, 95% CI: 1.07–3.06, *p* = .027; Figure 3). At the group level, over‐wintering survival was lower in bolder groups than shyer groups (*r* = −.807, *p* = .015; Figure 4a, Table S3), but no relationship was found between survival and boldness in bold or shy group members (Table S3). Moreover, no correlation was found between boldness and over‐wintering reproduction (Figure 4b, Table S3). We also detected no relationships between group composition and any survival or reproduction parameters (Figure 4c,d, Table S3).

## 4 DISCUSSION

The present study is the first to demonstrate the repeatability of bold–shy personality types in wild Mongolian gerbils. Our results also provided evidence of links between the boldness personality trait and food hoarding, as well as over‐wintering survival in captivity. Consistent with our prediction, at the individual and/or social levels, bolder gerbils tended to hoard later, less frequently, and with shorter foraging bouts. They also hoarded less food than shy gerbils, suggesting that shy gerbils and social groups may consistently hoard more food resources during the food hoarding season. Moreover, we found that the potential predator risks will affect gerbils' hoarding choices. Both bold and shy gerbils showed increased hoarding latency in food patches with higher potential predation risk. Additionally, the duration of each hoarding bout was shorter in bold gerbils, suggesting that the potential predation context played a significant role in their hoarding decisions. Finally, at both the individual and group levels, shy gerbils exhibit higher over‐wintering survival rates. However, there is no significant correlation between boldness and over‐wintering reproduction. In addition, the over‐wintering survival or reproduction of the group was not affected by group composition.

In some central theories, bolder individuals exhibit a faster pace of life characterized by rapid growth, early reproduction, and short longevity based on intrinsic states involving lower energy reserves, high energy processing ability, and so on (Réale et al., 2010; Sih et al., 2015). Therefore, bolder individuals should exhibit various behaviors to support their fast pace of life, such as being willing to take risks, foraging more frequently, and spending more time foraging to satisfy their current high‐energy demands (Dammhahn et al., 2018). However, these theoretical predictions were not generally accurate. A recent meta‐analysis indicated that the covariates between boldness and fitness may vary depending on the context, and the relationship between boldness and survival may also be inconsistent (Haave‐Audet et al., 2022; Moiron et al., 2020). Therefore, the covariation between boldness and behavior may be generated by the trade‐off of environmental risks. Hoarding food is a distinct foraging behavior that differs from general foraging because it involves an investment in future fitness rather than current fitness. In a previous study, Liu et al. (2011) showed that hoarding more food could increase the over‐wintering survival rate of gerbils. This suggests that food hoarding behavior is generated from a trade‐off in future starvation risks. Therefore, we argue that gerbils exhibit personality‐specific food hoarding behaviors, which are caused by their fitness trade‐offs during the over‐wintering season.

From an evolutionary perspective, residual reproductive value, that is, future fitness expectations, should result in systematic differences in risk‐taking behavior. Individuals with high future expectations (who have much to lose) should be more risk‐averse than individuals with low expectations (Wolf et al., 2007). Some studies have found that bolder individuals tend to have higher reproductive success (Bonnot et al., 2018), while shyer individuals tend to live longer (Careau et al., 2010). This suggests a trade‐off between longevity and reproduction based on bold and shy personalities. Previous studies by Liu et al. (2017) have shown individual differences in the expected lifespan of Mongolian gerbils. The average lifespan of gerbils is approximately 5 months, but some gerbils can survive for up to 30 months in their natural environment. According to the POLS hypothesis, shy gerbils are expected to have a longer life history. The food hoarding strategies of shy gerbils may have been driven by their long‐term energy requirements and future fitness benefits. Shy gerbils exhibited faster and more frequent hoarding and were more willing to take risks when hoarding food to maximize their over‐wintering survival. In contrast, bold gerbils may have made decisions based on their current energy state and immediate benefits due to their faster pace of life, characterized by rapid growth, early reproduction, and short longevity. These differences in the performance of food hoarding strategies by Mongolian gerbils also illustrate the evolutionary consequences of personality in life history, which are caused by the trade‐off between current and future survival expectations.

The state‐behavior feedbacks might provide another explanation for the correlation between boldness and hoarding behavior in Mongolian gerbils. The game theory of state dependency and behavioral specialization proposes that individuals may have personality‐specific energy demands, physiological characteristics, or environmental contexts (e.g., food availability, temperature, or predator risk) (Biro & Stamps, 2010). Some studies have shown that bolder rooks (*Corvus frugilegus*) and bolder fallow deer (*Dama dama*) tend to forage more than their shyer conspecifics (Bergvall et al., 2011; Jolles et al., 2013). This may be because bolder individuals are likely to have a higher body mass and metabolic rate, which requires them to consume more energy to maintain a higher metabolic rate. This implies that bold individuals are likely to exert more effort during foraging (Réale et al., 2010). A recent meta‐analysis found that although behaviors, life history, and physiology were correlated, these correlations were not always consistent with the predictions of the POLS hypothesis (Royauté et al., 2018). Some studies found that bolder individuals had a higher metabolic rate, such as the round goby (*Neogobius melanostomus*) (Behrens et al., 2020; Myles‐Gonzalez et al., 2015) and western stutter‐trilling crickets (*Gryllus integer*) (Krams et al., 2017). However, the opposite was also shown in other empirical studies, such as in the greater white‐toothed shrew (*Crocidura russula*) (Oliveira et al., 2020). In wild eastern chipmunks (*Tamias striatus*), individuals that are more explorative and aggressive (traits theoretically positively correlated with boldness) were found to have a lower daily energy expenditure (Careau et al., 2015). The correlation between boldness and higher metabolism seems to be inconsistent across species. More diverse connections should exist between personality, individual state‐behavioral strategies, and fitness. Actually, our laboratory study demonstrated that the daily energy expenditure was higher in shyer gerbils than in bolder gerbils (L. Gan, unpublished data). Consequently, we consider that the varying energy demands or metabolic rates of wild gerbils might be associated with their personality‐specific food hoarding strategies. The lower probability of an energy shortfall or the lower energy demand state of bold gerbils may explain their reduced hoarding demand. Thus, more detailed experiments and procedures are required to explore this hypothesis further under realistic ecological conditions.

The role‐related differences in group cooperative hoarding behavior may be another reason for the variations in hoarding activities. A study of barnacle geese (*Branta leucopsis*) showed that shyer individuals exhibited foraging behavior more often, but these shy individuals exhibited feeding behavior less frequently (Kurvers et al., 2010). These findings indicate that variations in foraging behavior may not be solely due to feeding requirements but rather related to differences in foraging strategies. Indeed, previous studies have shown that gerbils are resource‐defending social rodents primarily focused on defending food resources (Ebensperger, 2001). They are also cooperative hoarding species, with most members participating in hoarding activities, and some individuals are actively involved in defending their territories when hoarding food (Agren et al., 1989b; Liu et al., 2005). Thus, we suggest that bold gerbils may play the role of alerting or protecting resources, while shy gerbils exhibit a direct food hoarding role. Regrettably, we could not record enough data on the behaviors of different classes of gerbils during our food hoarding experiments. Thus, a positive correlation between boldness and foraging behavior, as predicted by the POLS hypothesis, could not be observed in this study. This relationship might have been obscured by the social food storage division decisions made by the social rodents. Of course, a positive correlation between boldness in gerbils and their foraging behavior may exist in other environmental contexts, such as food availability in a fluctuating environment or in the presence of intraspecific competitors. Further research is needed to evaluate these potential correlations in the future.

Moreover, our results showed that both bold and shy gerbils will reduce hoarding in high‐risk food patches. This suggests that the environmental context may also determine how personality affects animals' hoarding decisions (Dall et al., 2004). Some studies have shown that indirect predation risk cues play a crucial role in regulating foraging behavior. For example, research has demonstrated how vegetation cover influences the foraging strategies of the fat sand rat (*Psammomys obesus*), a species that predominantly forages in shrubs and hoards food in open terrace environments (Tchabovsky et al., 2001). In the golden spiny mouse (Acomys russatus), the giving‐up density was correlated with microhabitat. Specifically, the giving‐up density was higher in a food patch with no overhead cover compared to a food patch with overhead shelter (Levy et al., 2016). Similarly, in the present study, our findings indicated that hoarding strategies were dependent on indirect predation risk. This dependency may have been related to variations in the trade‐off between hoarding frequency or time and potential predation risk among individuals. Furthermore, these strategies could have been influenced by an individual's personality traits.

Finally, we found no effect of boldness on over‐wintering reproduction (Table S3, Figure 3b). On the one hand, the restriction on reproduction by gerbils during the over‐wintering period may have been caused by seasonal food shortages and increased survival costs. In general, the availability of food is crucial for seasonal reproduction by rodents (Taylor & Calaby, 2004). In northern latitudes, rodents are likely to experience food shortages and increased energy demands in the winter (Jackson et al., 2001; Merritt, 1995; Wang & Wang, 1996). In previous studies, Liu et al. (2007, 2013) showed that Mongolian gerbils typically experience two distinct seasons in terms of their annual life history. There is a reproduction season from March to August when plenty of food is available for gerbils, and a breeding lull season in autumn and winter when gerbils mainly feed on their hoarded food. Therefore, we argue that seasonal ecological contexts could have masked the difference in over‐wintering reproduction between bold and shy individuals, which may represent alternative strategies with environmentally dependent adaptive value. On the other hand, considering the low reproductive output to begin with, we also cannot discount the possibility that the small sample size could have obscured any significant effects resulting from seasonal fluctuations in the population. Furthermore, the small sample size on which the “reproduction” variable is based may mask any significant associations and/or effects with the other variables (personality score, foraging strategy). Moreover, a new framework based on a field study of colonial spiders (*Cyrtophora citricola*) showed that food sharing in a group plays a critical role in reproduction (Grinsted et al., 2019). Thus, a potential correlation between boldness and over‐wintering reproduction could have been masked by boldness measures in other females in the group, and additional research is required to clarify this issue.

In conclusion, our results demonstrate that boldness affects hoarding behavior and over‐wintering survival at both the individual and group levels in Mongolian gerbils. Both bold and shy gerbils were able to assess the risk of predation in a food patch. We found no support for a positive link between boldness and foraging effort. Surprisingly, shyer gerbils actually expended more effort in foraging compared with bold gerbils during the food hoarding season. Additionally, their over‐wintering survival rate was greater than that of bold gerbils. Though boldness types in this study were dichotomized according to a threshold value, this method of dichotomization may lead to biased effect size estimates and increase false positive rates. However, considering the significant difference in boldness scores between bold and shy individuals in this study, we believe this bias could be negligible to some extent. Our results indicated that personality‐specific foraging behavior was influenced by life history trade‐offs. Moreover, it was also shown that group boldness did not affect over‐wintering reproduction. This effect may have been obscured by suppressed reproduction during the winter or by the small sample size of offspring and surviving adult gerbils in our experiments. Furthermore, the differences in survival rates among various personality types may impact the personality composition within spring breeding groups, potentially influencing spring breeding and ultimately affecting the population dynamics of Mongolian gerbils. Our findings suggest that the hoarding behaviors of gerbils and their connection with personality traits may play a crucial role in their life history and ecology. A variety of factors, such as personality, individual characteristics, and environmental conditions, may collectively influence the hoarding strategies that species adopt in nature.

Overall, our results demonstrated that boldness affected hoarding behavior but also over‐wintering survival in these wild group‐living rodents, and thus, the hoarding behaviors of gerbils and their links with personality may be important for their life history and ecology.

## AUTHOR CONTRIBUTIONS

**Lin Gan:** Conceptualization (equal); data curation (equal); formal analysis (lead); investigation (equal); writing – original draft (lead). **Shuang‐Jie Tian:** Investigation (equal). **De‐Hua Wang:** Conceptualization (equal); funding acquisition (equal); project administration (equal); resources (equal); supervision (equal); writing – review and editing (equal). **Wei Liu:** Conceptualization (equal); funding acquisition (equal); project administration (equal); resources (equal); supervision (equal); writing – review and editing (equal).

## CONFLICT OF INTEREST STATEMENT

The authors have no conflicts of interest to declare.

## Supporting information


Tables S1–S3.

## Data Availability

The data that support the findings of this study are openly available in Dryad at: https://datadryad.org/stash/share/U‐3875gg6WkLRM7v9a6B‐wZ7xkaoOiR‐l_YX5DClGJ4.
