# Supplementary material for: Boldness suppresses hoarding behavior in food hoarding season and reduces over‐wintering survival in a social rodent
Source: Ecol Evol. 2024 Apr 9;14(4):e11252. doi: 10.1002/ece3.11252 (PMC11004661; doi:10.1002/ece3.11252)
Supplement: Supplementary file 1 — Tables S1–S3. [file ECE3-14-e11252-s001.docx]

**Supplementary material**

**Table S1** Effects of individual (n = 24) boldness (bold and shy), food patch type (UC and NC), and sex (male and female) on foraging behavior of Mongolian gerbils (*Meriones unguiculatus*) observed in field enclosures from mid-September 2019 to mid-October 2019 in Taibusi Qi, Inner Mongolia, China

| Behavior type |  | Posterior mean | Lower 95% CI | Upper 95% CI | pMCMC |
| --- | --- | --- | --- | --- | --- |
| Foraging latency | | | | | |
|  | (Intercept) | **5415.5** | **3687.9** | **7488.6** | **0.018** |
|  | Personality_shy_ | **-2461.0** | **-4351.9** | **-695.8** | **0.008** |
|  | Food patch_UC_ | **-1634.9** | **-2794.1** | **-505.0** | **0.014** |
|  | Sex_male_ | -383.5 | -2512.6 | 1644.5 | 0.696 |
|  | Personality_shy_×Food patch_UC_ | 1532.3 | -19.0 | 3059.3 | 0.054 |
|  | Personality_shy_×Sex_male_ | 1311.3 | -1406.1 | 4015.5 | 0.330 |
|  | Food patch_UC_×Sex_male_ | 776.6 | -1078.2 | 2463.6 | 0.390 |
|  | Personality_shy_×Food patch_UC_×Sex_male_ | -1299.5 | -3444.9 | 1017.9 | 0.286 |
| Foraging frequency | | | | | |
|  | (Intercept) | 17.53 | -5.98 | 39.38 | 0.100 |
|  | Personality_shy_ | **32.07** | **8.88** | **56.18** | **0.008** |
|  | Food patch_UC_ | 14.50 | -3.06 | 29.54 | 0.096 |
|  | Sex_male_ | 11.18 | -14.66 | 39.73 | 0.426 |
|  | Personality_shy_×Food patch_UC_ | -13.26 | -32.89 | 10.46 | 0.252 |
|  | Personality_shy_×Sex_male_ | -22.99 | -61.77 | 11.25 | 0.226 |
|  | Food patch_UC_×Sex_male_ | -18.88 | -42.20 | 8.92 | 0.162 |
|  | Personality_shy_×Food patch_UC_×Sex_male_ | 19.14 | -14.74 | 51.95 | 0.296 |
| Duration of each foraging bout | | | | | |
|  | (Intercept) | **24.28** | **13.35** | **47.05** | **0.018** |
|  | Personality_shy_ | **15.57** | **2.67** | **31.56** | **0.040** |
|  | Food patch_UC_ | **15.47** | **0.71** | **30.97** | **0.038** |
|  | Sex_male_ | -3.51 | -21.47 | 20.38 | 0.734 |
|  | Personality_shy_×Food patch_UC_ | **-23.27** | **-41.56** | **-5.18** | **0.012** |
|  | Personality_shy_×Sex_male_ | -14.01 | -41.01 | 9.43 | 0.266 |
|  | Food patch_UC_×Sex_male_ | 1.24 | -24.17 | 24.46 | 0.912 |
|  | Personality_shy_×Food patch_UC_×Sex_male_ | 16.96 | -11.62 | 47.88 | 0.294 |

Posterior means, 95% confidence intervals, and probability values (pMCMC) are presented. Model estimates are shown in bold when the confidence intervals do not overlap 0.

**Table S2** Overall survival and reproduction by Mongolian gerbils (*Meriones unguiculatus*) in mid-September 2019 and May 2020 in Taibusi Qi, Inner Mongolia, China

| Date | Survival | | Reproduction | |
| --- | --- | --- | --- | --- |
|  | Male | Female | Male | Female |
| September of 2019 | 9 | 15 | 0 | 0 |
| May of 2020 | 5 | 9 | 14 | 13 |

**Table S3** Multiple correlations between group boldness, group composition, over-wintering survival, and reproduction in Mongolian gerbils (*Meriones unguiculatus*) Over-wintering survival and reproduction were determined in field enclosures in May 2020 in Taibusi Qi, Inner Mongolia, China.

|  | Survival | Bold survival | Shy survival | Reproduction |
| --- | --- | --- | --- | --- |
| Boldness score | 0.807^*^ | 0.088 | 0.354 | 0.495 |
| Group composition | -0.259 | 0.209 | 0 | -0.042 |

Group composition represents the proportion of bold gerbils in a group. ^*^*p* < 0.05.
